# Optimal Chest Compression Rate and Compression to Ventilation Ratio in Delivery Room Resuscitation: Evidence from Newborn Piglets and Neonatal Manikins

**DOI:** 10.3389/fped.2017.00003

**Published:** 2017-01-23

**Authors:** Anne Lee Solevåg, Georg M. Schmölzer

**Affiliations:** ^1^The Department of Pediatric and Adolescent Medicine, Akershus University Hospital, Lørenskog, Norway; ^2^Centre for the Studies of Asphyxia and Resuscitation, Neonatal Research Unit, Royal Alexandra Hospital, Edmonton, AB, Canada

**Keywords:** newborn, cardiopulmonary resuscitation, chest compression, manikins, piglet

## Abstract

Cardiopulmonary resuscitation (CPR) duration until return of spontaneous circulation (ROSC) influences survival and neurologic outcomes after delivery room (DR) CPR. High quality chest compressions (CC) improve cerebral and myocardial perfusion. Improved myocardial perfusion increases the likelihood of a faster ROSC. Thus, optimizing CC quality may improve outcomes both by preserving cerebral blood flow during CPR and by reducing the recovery time. CC quality is determined by rate, CC to ventilation (C:V) ratio, and applied force, which are influenced by the CC provider. Thus, provider performance should be taken into account. Neonatal resuscitation guidelines recommend a 3:1 C:V ratio. CCs should be delivered at a rate of 90/min synchronized with ventilations at a rate of 30/min to achieve a total of 120 events/min. Despite a lack of scientific evidence supporting this, the investigation of alternative CC interventions in human neonates is ethically challenging. Also, the infrequent occurrence of extensive CPR measures in the DR make randomized controlled trials difficult to perform. Thus, many biomechanical aspects of CC have been investigated in animal and manikin models. Despite mathematical and physiological rationales that higher rates and uninterrupted CC improve CPR hemodynamics, studies indicate that provider fatigue is more pronounced when CC are performed continuously compared to when a pause is inserted after every third CC as currently recommended. A higher rate (e.g., 120/min) is also more fatiguing, which affects CC quality. In post-transitional piglets with asphyxia-induced cardiac arrest, there was no benefit of performing continuous CC at a rate of 90/min. Not only rate but duty cycle, i.e., the duration of CC/total cycle time, is a known determinant of CC effectiveness. However, duty cycle cannot be controlled with manual CC. Mechanical/automated CC in neonatal CPR has not been explored, and feedback systems are under-investigated in this population. Evidence indicates that providers perform CC at rates both higher and lower than recommended. Video recording of DR CRP has been increasingly applied and observational studies of what is actually done in relation to outcomes could be useful. Different CC rates and ratios should also be investigated under controlled experimental conditions in animals during perinatal transition.

## Introduction

**Figure 1:**
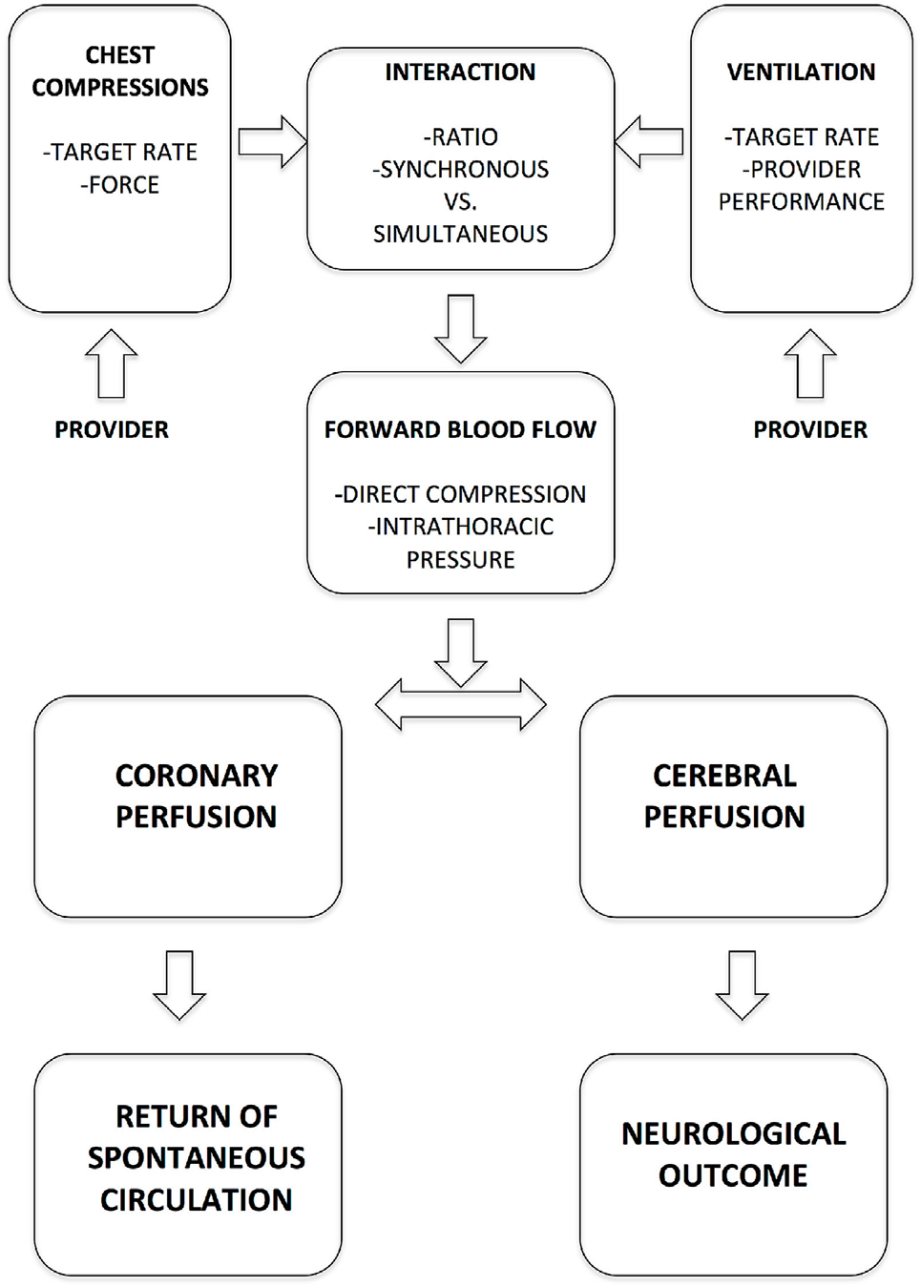
**Key determinants of chest compression effectiveness in delivery room cardiopulmonary resuscitation**.

Observational data indicate that prolonged cardiopulmonary resuscitation (CPR) in the delivery room (DR) is associated with poor survival and neurologic outcomes (1). High quality chest compressions (CC) improve cerebral and myocardial perfusion. Improved cerebral perfusion ensures brain cell survival during CPR, whereas enhanced myocardial perfusion increases the likelihood of a fast return of spontaneous circulation (ROSC). Thus, optimizing CC quality may improve outcomes both by preserving cerebral blood flow during cardiac arrest and by reducing recovery time. The effectiveness of CC is influenced by (i) CC rate, (ii) CC to ventilation (C:V) ratio, and (iii) applied force, which are all influenced by the CC provider (Figure 1). Thus, besides CPR mechanics and mathematical modeling, educational, emotional, and physical aspects of provider performance need to be considered when addressing the optimal CC algorithm. Ultimately, the physiological effects in the infant determine the most optimal CC intervention.

In animals with ventricular fibrillation (VF) cardiac arrest, adequately oxygenated blood can be circulated with CC for up to 4–6 min even without assisted ventilation (2). In addition, pediatric and adult animal data demonstrate better coronary perfusion pressures (CPP) with less CC interruption (3–6). Thus, more uninterrupted CC in a series is generally regarded to be beneficial. However, because cardiovascular collapse in the DR is almost invariably due to hypoxia, neonatal resuscitation guidelines recommend a 3:1 C:V ratio (7). CCs should be delivered at a rate of 90/min synchronized with ventilations at a rate of 30/min to achieve a total of 120 events/min (7). There is a lack of scientific evidence to support this standard of care in neonatal CPR, and the unique physiology of perinatal transition as well as cerebral and cardiovascular immaturity call for special considerations in DR CPR. However, investigations of alternative CC interventions in the critically ill human neonate are ethically challenging. Also, the infrequent occurrence of extensive CPR in the DR makes randomized controlled trials difficult to perform. Thus, many biomechanical aspects of CC have been studied in animal and manikin models. In such models, operator performance can also be assessed in a systematic fashion. Because of physical similarities between piglets and human infants including broader chest compared to other non-primate species, and similar chest size and stiffness (8), the porcine model of neonatal CPR has been extensively studied. The aim of this review is to provide an overview about the current knowledge of optimal CC rate and C:V ratio during neonatal CPR.

A summary of studies investigating different CC rates and C:V ratios in neonatal CPR is presented in Table 1.

**Table 1:** **Summary of studies exploring different CC rates and C:V ratios in neonatal resuscitation**.

Reference	Design	Subjects	Interventions	Outcomes	Conclusion
Solevåg et al. (13)	Randomized controlled animal trial	Piglets 1–3 days of age weight 1.7–2.4 kg (*n* = 32)	Asphyxiated piglets were randomized to 3:1 C:V CPR or CCaV CPR (CC rate 90/min)	Time to ROSC was similar for 3:1 C:V CPR and CCaV CPR; *p* = 0.84	Overall recovery may be similar, but CCaV might impair myocardial perfusion compared to 3:1 C:V CPR
				Post mortem analysis of left ventricle lactate was increased in the CCaV group	

Solevåg et al. (11)	Randomized controlled animal trial	Piglets 12–36 h old (*n* = 32)	Asphyxiated piglets were randomized to receive a C:V ratio of 3:1 or 9:3	Time to ROSC (median and interquartile range) was 150 (115–180) s vs. 148 (116–195) s for 3:1 and 9:3, respectively (*p* = 0.74). There were no differences in diastolic blood pressure (DBP) during compression cycles or in markers of hypoxia and inflammation	The C:V ratio 9:3 was not better than 3:1

Solevåg et al. (12)	Randomized controlled animal trial	Piglets 12–36 h old (*n* = 22)	Asphyxiated piglets were randomized to receive a C:V ratio 3:1 or 15:2	Mean (SD) increase in DBP (mmHg) during compression cycles was significantly higher with 15:2 than 3:1 C:V [7.1 (2.8) vs. 4.8 (2.6)]. Median (interquartile range) time to ROSC for the 3:1 group was 150 (140–180) s, and 195 (145–358) s for the 15:2 group	The C:V ratio 15:2 is not better than 3:1 in neonatal resuscitation

Dannevig et al. (9)	Randomized controlled animal trial	Piglets 12–36 h old (*n* = 94)	Asphyxiated piglets were resuscitated with a C:V ratio of 3:1, 9:3, or 15:2	Interleukin-6 (IL-6), tumor necrosis factor-α (TNF-α), and S100 in CSF, and gene expression of matrix metalloproteinases (MMPs), intercellular adhesion molecule-1 (ICAM-1), caspase 3, IL-6 and TNF-α in hippocampus and frontal cortex tissue were similar across C:V groups	Higher C:V ratios did not change the brain inflammatory response compared with the 3:1 C:V ratio

Dannevig et al. (17)	Randomized controlled animal trial	Piglets 12–36 h old (*n* = 72)	Asphyxiated piglets were resuscitated with a C:V ratio of 3:1, 9:3, or 15:2	IL-8 and TNF-α in BAL fluid and MMP2, MMP9, ICAM-1, and TNFα in lung tissue were similar across C:V groups	Higher C:V ratios did not change the lung inflammatory response compared with the 3:1 C:V ratio

Boldingh et al. (30)	Randomized crossover manikin trial	Doctors, nurses, and midwifes (*n* = 34)	5 min CPR, with either a 3:1 C:V ratio or CCaV (CC rate 120/min). All participants performed all interventions in a randomized order	The CC proportion with adequate depth was 90.5% for the 3:1 C:V ratio and 60.1% in CCaV	CCaV is more exhausting than a 3:1 C:V ratio
				CCaV resulted in a greater increase in rescuer heart rate and mean arterial blood pressure, and perceived fatigue, compared to 3:1 C:V CPR	

Li et al. (28)	Randomized crossover manikin trial	Neonatologists, neonatal-perinatal fellows, neonatal nurse practitioners, and registered nurses (*n* = 30)	10 min CPR with a 3:1 C:V ratio, CCaV-90 (CCaV at a 90/min rate), and CC at a rate of 120/min (CCaV-120) (CCaV at a 120/min rate). All participants performed all interventions in a randomized order	Peak CC pressure decreased significantly after 156, 96, and 72 s in the 3:1, CCaV-90, and CCaV-120 groups, respectively	3:1 C:V CPR was the least fatiguing and the most preferred method

Boldingh et al. (29)	Randomized crossover manikin trial	Doctors, nurses, midwives, and last-year medical students (*n* = 84)	2 min of CPR with C:V ratios 3:1, 9:3, or 15:2—and CCaV (CC rate 120/min). All participants performed all interventions in a randomized order	3:1 C:V and 9:3 C:V were comparable in terms of CC and ventilation dynamics	The results of the study support the currently recommended 3:1 C:V ratio
				The 15:2 C:V ratio resulted in less ventilation vs. the 3:1 C:V ratio	
				The mean CC depth with CCaV vs. the 3:1 method was 32.7 vs. 34.6 mm (*p* < 0.001)	
				There was a significant decrease in CC depth from baseline after 60 s (*p* = 0.025) with CCaV	
				The two-person CRP coordination was rated easiest with the 3:1 C:V ratio	

Solevåg et al. (31)	Randomized crossover manikin trial	Two medical students	Ten times 2 min 3:1 C:V CPR, 9:3 C:V, and 15:2 C:V—and CCaV (CC rate 120/min) were performed in a randomized order	Minute ventilation in mL/kg was significantly lower at the C:V ratios 9:3 [140 (134–144)] and 15:2 [77 (74–83)] vs. 3:1 [191 (183–199)]	Higher C:V ratios than 3:1 compromised ventilation

*CC, chest compression; CPR, cardiopulmonary resuscitation; CCaV, continuous chest compression and asynchronous ventilation; ROSC, return of spontaneous circulation; CSF, cerebrospinal fluid; BAL, bronchoalveolar lavage*.

### Piglets

#### Hemodynamic Effects of CC

##### Biomarkers of Cerebral Perfusion

As an indirect measure of cerebral “well-being” during CPR, Dannevig et al. (9) measured inflammation markers [interleukin-6 (IL-6), tumor necrosis factor-α (TNF-α), and S100] in cerebrospinal fluid (CSF), and gene expression of matrix metalloproteinases (MMPs), intercellular adhesion molecule-1 (ICAM-1), caspase 3, IL-6, and TNF-α in hippocampus and frontal cortex tissue. They found that the CSF cytokine concentrations and tissue quantitative real-time PCR in the three C:V ratio groups 3:1, 9:3, and 15:2 were not different (9). This might indicate that in asphyxia, higher C:V ratios do not improve cerebral perfusion in asphyxiated infants and contrasts to the improved neurological outcomes when CC are not being interrupted in VF cardiac arrest (10).

##### Myocardial Perfusion

Diastolic blood pressure (DBP) is a proxy for myocardial perfusion during CPR. Solevåg et al. reported similar DBP between 3:1 and 9:3 C:V ratio CPR in a piglet model of asphyxia-induced cardiac arrest (11). Interestingly, using a 15:2 C:V ratio resulted in a higher mean (SD) DBP of 7.1 (2.8) vs. 4.8 (2.6) mmHg during standard 3:1 C:V CPR (*p* = 0.004) (12). Unfortunately, time to ROSC was similar between 15:2 and 3:1 C:V CPR. In a similar study (13), CCaV had similar DBP compared to 3:1 C:V CPR. Post mortem analysis of left ventricle lactate was increased in the CCaV group, which might indicate either anaerobic metabolism or physical trauma, or a combination of both (13).

#### Physical Trauma/Inflammation

There is a lack of knowledge about lung injury resulting from neonatal CPR. Clinical studies reported an increase in inflammatory markers in blood early after thoracic trauma (14–16). Dannevig et al. (17) analyzed IL-8 and TNF-α in bronchoalveolar lavage (BAL) fluid and MMP2, MMP9, ICAM-1, and TNFα in lung tissue of asphyxiated piglets after CPR. They observed no differences in IL-8 or gene expression in lung tissue between piglets resuscitated with a C:V ratio of 3:1 or 9:3. However, median (IQR) TNF-α was 1.26 (0.92–1.55) pg/ml in the 9:3 group vs. 0.88 (0.67–1.28) pg/ml in the 3:1 group (*p* = 0.047). Furthermore, inflammatory markers in BAL and lung tissue were increased in piglets where CC were initiated after a 30-s ventilation period compared to 60 and 90 s suggesting that a higher total number of CC during CPR might negatively influence outcome through inflammatory pathways (17).

#### Epinephrine

Through its α-adrenergic effects, epinephrine mediates peripheral vasoconstriction while forward flow is generated with CC (18), thus elevating the aortic pressure and CPP in pigs (19). Epinephrine has been thought to be essential for ROSC in severe perinatal compromise (20). However, recent experimental data in lambs (21) and piglets (11, 12, 22, 23) are conflicting. Pytte et al. (24) speculated that the hemodynamic effects of epinephrine depend on CC quality, which might explain the conflicting results in different animal models. In asphyxiated piglets, Linner et al. (23) and McNamara (25) found that ROSC could be achieved with CC alone without epinephrine, whereas Solevåg et al. (11, 12) reported that piglets almost invariably required epinephrine for ROSC. Similar findings were made in lambs by Sobotka et al. (21). One important difference between the study protocols was that Linner et al. (23) targeted CC to a mean arterial blood pressure (MAP) of 35–40 mmHg, whereas Solevåg et al. (11, 12) targeted the CC to a MAP of 20 mmHg. McNamara (25) did not specify a pressure target but aimed to compress the chest by one-third of the anteroposterior diameter. Lambs have different chest geometry than piglets, and the two models are not easily comparable in terms of CC quality (26).

### Manikins

#### CC Rate

Neonatal CPR guidelines recommend 120 events/min, which comprise 90 CC and 30 inflations (7). CC duty cycle is the CC duration divided by total cycle time. Increasing the CC rate increases the effective duty cycle by decreasing the total cycle time provided that the CC duration remains constant (8). In fact, mathematical models suggest that the optimal CC rate in newborn infants should exceed 120/min (27). Manikin studies have investigated the feasibility of performing neonatal CC at different rates. Li et al. (28) showed that even though it was possible for neonatal staff to perform continuous CC at rates of 90 and 120/min, a significant decay in CC pressure occurred after 96 and 72 s, respectively. In contrast, when performing standard 3:1 C:V CPR, significant decay occurred only after 156 s. This means that good quality CC might be maintained for more than twice as long with 3:1 C:V CPR compared with uninterrupted CC at a rate of 120/min (CCaV-120). In addition, the 3-min CC depth decline was 50% if CC was performed at a rate of 120/min vs. 30% at a rate of 90/min (28). Similarly, Boldingh et al. (29) found that the CC depth was lower in CCaV-120 compared with the 3:1 C:V ratio method, with a significant decline in CC depth from baseline after 60 s in CCaV-120 vs. no significant decline after 120 s in 3:1 C:V CPR. Boldingh et al. (29) did not investigate continuous CC at a rate of 90/min, but quite unanimously, studies indicate that rescuer fatigue is more pronounced when CC are performed continuously compared to when a pause is inserted after every third CC as currently recommended (28, 29). This is further supported by the preference of resuscitators of the 3:1 C:V ratio over CCaV (28–30), and the two-person coordination is easier with the 3:1 C:V ratio (29). On the other hand, CCaV has been shown to improve minute ventilation during manikin CPR (29, 31).

#### Educational, Emotional, and Physical Aspects of Operator Performance

##### Educational

The International Liaison Committee on Resuscitation stated, in their 2015 treatment recommendations (7), that despite very low quality evidence in favor of the 3:1 C:V ratio, they chose to retain the recommendation for educational reasons, as the value of consistency of the algorithm was thought to be significant. There is a lack of data demonstrating educational advantages of alternative C:V ratios.

##### Emotional

Rescuers’ perceptions of self-efficacy may influence skills acquisition and retention. “Perceived control,” determined by knowledge, competencies, skills, ability, and experience, is a determinant of behavior (32). Education about CC dynamics and physiology, as well as practical CC training can therefore both through knowledge and skills acquisition, and also through enhanced perceived control, positively influence CC performance. Perceived control in the DR may also alleviate the stress experienced during CPR. Some investigators have tried to incorporate the element of stress in simulated CC scenarios (29), but practicing CC in an artificial simulated environment can never replicate the stress of real-life CPR.

##### Physical

Boldingh et al. (30) assessed heart rate (HR), MAP, and respiratory rate of rescuers as measures of physical fatigue during CPR with either 3:1 C:V CPR or CCaV-120. In addition, they investigated whether body mass index (BMI) and weekly physical activity influenced CC performance. CCaV-120 resulted in a greater increase in rescuer HR and MAP compared to a 3:1 C:V ratio. In agreement with this, continuous CC were perceived as being more fatiguing. Weekly physical activity and BMI did not seem to influence CC performance.

## Discussion

In asphyxiated newborn piglets, harm caused by the trauma of CC may counterbalance the beneficial effects of more CC on hemodynamics seen in pediatric and adult animals. The reasons for this are at least twofold: (1) Dean et al. (33) speculated that “standard” CPR is already quite effective in animals with small and compliant chests. Thus, increasing CC rates may not have additive effects in infant CPR, (2) as the cause of profound bradycardia or cardiac arrest in the DR is almost invariably asphyxia, the inflammatory cascade is already highly activated at the time CPR with CC is commenced. The additional trauma of performing CC may add to the injury of vital organs and result in a poor outcome.

The asphyxial etiology of arrest in the DR is of course complicating the picture in a multitude of other ways. Piglet studies suggest that in severe asphyxia, the systemic vascular resistance (SVR) initially increases and then decreases (34). Almost concurrently with the drop in SVR, the MAP declines (34). Aaltonen et al. (34) speculated that this drop in MAP could not be explained by a reduced left ventricular output. This is supported by results from Li et al. (35) that cardiac output in asphyxiated newborn piglets after CPR was not different from control piglets. Thus, a more likely reason for hypotension in the asphyxic neonate is the profound acidosis and lack of substrate (ATP). ATP is required for maintaining vascular tone, and sustained asphyxia will eventually result in maximal vasodilation (36). Thus, blood flow generated with CC preferentially goes through a dilated aorta and into the peripheral circulation rather than into the smaller, higher-resistance coronary arteries (36). This is a plausible explanation for why attempts at increasing coronary perfusion by increasing the C:V ratio or even CC rate has not been successful in models of perinatal asphyxia. CCaV has not proven to be of benefit in asphyxiated piglets, unless they are combined with a sustained inflation (35, 37). Manikin studies support that continuous CC and asynchronous standard positive pressure ventilation might not provide benefit. In fact, this method may result in suboptimal CC quality (28, 29). Rescuers typically prefer standard 3:1 C:V CPR (28, 29).

### Knowledge Gaps

#### Effects of CC in the Absence of a Functioning Cerebral Autoregulation

As cerebral oxygen delivery depends on cerebral blood flow and arterial oxygen content, cerebral oxygen delivery is maintained during hypoxia by an increase in cerebral blood flow (38). However, even brief hypoxia (20 min) results in impairment of cerebral blood flow autoregulation, especially during hypotension episodes in animal models (39, 40). Rosenberg (41) demonstrated in newborn lambs that after asphyxia, the lambs exhibited a markedly impaired vasodilation in response to hypoxia, and no cerebral vasodilation with systemic hypotension (41), quite different from adult models where cerebral autoregulation is intact after asphyxia (42). These differences between newborns and adults could be the result of maturation and should be taken into account when attempts are made to establish the best CC strategy in asphyxiated infants. Since asphyxia causes an impairment of cerebral autoregulation in the newborn infant, the risk of brain damage resulting from fluctuations in blood pressure caused by CC and inotropes should not outweigh the benefits of improved coronary and systemic perfusion. The focus of CC research has been on systemic and coronary hemodynamics with the primary endpoint often being ROSC. Even though neurological adverse effects of epinephrine in preterm infants have been described (43, 44), less attention has been given to the effects of CC alone on cerebral circulation in asphyxiated term infants.

#### Preterm Infants

Preterm infants requiring CPR with CC have a high mortality and many of the infants that survive develop neurodevelopmental impairment (45). A fragile germinal matrix and poor cerebral autoregulation in preterm infants predispose for intraventricular hemorrhage and may be among the reasons why preterm infants potentially need different CPR strategies compared to term or near-term infants.

#### Oxygen Fractions

One of the proposed mechanisms behind the impaired cerebral autoregulation after asphyxia is vascular endothelial injury secondary to oxygen-free radical production during reperfusion (46). Hyperoxia during CPR in perinatal asphyxia is detrimental in a multitude of other ways. This has been extensively studied in animals and humans needing positive pressure ventilation. Far less is known about the balance between damage caused by hypoxia and hyperoxia when CC are needed (47).

#### Duty Cycle and Automated CC

Duty cycle, i.e., the duration of CC/total cycle time, is a known determinant of CC effectiveness in pediatric and adult models. In animals, the optimal CC rate and duty cycle differ between pediatric and adult models. This may also be the case in human children and adults and should thus be studied further. However, duty cycle cannot be controlled with manual CC. Mechanic CC in neonatal CPR has not been thoroughly explored.

#### CC Depth and Force

Most studies of CC efficacy in neonatal CPR have used CC depth as measure of CC quality. However, the CC depth that optimizes cerebral and myocardial perfusion remains unknown. Even though some studies have assessed leaning during CC administration, little attention has been given to over-compression of the chest, i.e., the slogan from adult CPR “push hard and fast” (48) has not been properly challenged in neonatal CPR. The depth of CC can be translated into compressive force, which is related to intrathoracic pressure (49). Feedback systems for both depth and force during neonatal CPR are insufficiently explored and deserve attention (50).

### Future Research Objectives

In real-life resuscitation, it is not uncommon that CC and assisted ventilations are being performed in a more or less asynchronous fashion. Evidence indicates that compliance with the algorithm is poor (51–53). Video recording of DR CRP has been increasingly applied and observational studies of what is actually done in relation to outcomes could potentially be useful.

The extensively used manikin models cannot replicate the complex mechanisms of antegrade blood flow during CPR. Manikins are one-dimensional with respect to the fact that blood flow during CC is a product of direct compression forces and the complex changes in intrathoracic pressure that occur. To reliably determine the effects of different CC interventions on regional and systemic hemodynamics, the use of transitioning animal models that more accurately replicate the newborn circulation with patent fetal shunts is required.

In conclusion, the very unique physiology of perinatal transition and asphyxia make DR resuscitation different from resuscitation at any time later in life. Most of what we know about CC dynamics from pediatric and adult basic and clinical research has to be challenged in appropriate neonatal models. Thus far, no CC study has been performed in a transitioning animal model, and is urgently needed.

## Author Contributions

AS and GS: substantial contributions to all of the following: (1) the conception and design of the manuscript, (2) drafting the manuscript or revising it critically for important intellectual content, and (3) final approval of the version to be submitted.

## Conflict of Interest Statement

The authors declare that the research was conducted in the absence of any commercial or financial relationships that could be construed as a potential conflict of interest.

## Abbreviations

CPR, cardiopulmonary resuscitation; DR, delivery room; CC, chest compressions; ROSC, return of spontaneous circulation; C:V ratio, chest compression to ventilation ratio; VF, ventricular fibrillation; CPP, coronary perfusion pressure; CSF, cerebrospinal fluid; CCaV, continuous CC with asynchronous ventilation; DBP, diastolic blood pressure; MAP, mean arterial blood pressure.
